# Environmental Contamination and Occupational Exposure of Algerian Hospital Workers

**DOI:** 10.3389/fpubh.2020.00374

**Published:** 2020-08-05

**Authors:** Eline Verscheure, Matteo Creta, Jeroen Vanoirbeek, Meziane Zakia, Taleb Abdesselam, Robin Lebegge, Katrien Poels, Radu-Corneliu Duca, Lode Godderis

**Affiliations:** ^1^Department of Public Health and Primary Care, Centre for Environment and Health, KU Leuven, Leuven, Belgium; ^2^Centre Hospitalo-Universitaire, Service Médicine du Travail, Université Abou Bekr Belkaid Tlemcen, Tlemcen, Algeria; ^3^TOXICOMED Research Laboratory, Faculty of Medicine, Université Abou Bekr Belkaid Tlemcen, Tlemcen, Algeria; ^4^National Health Laboratory (LNS), Unit Environmental Hygiene and Human Biological Monitoring, Department of Health Protection, Dudelange, Luxembourg; ^5^Idewe, External Service for Prevention and Protection at Work, Heverlee, Belgium

**Keywords:** antineoplastic drugs, chemotherapy, occupational hygiene, environmental monitoring, Africa

## Abstract

Guidelines are in place to assure limited occupational exposure to cytostatic drugs. Even though this has led to a reduction in exposure, several studies reported quantifiable concentrations of these compounds in healthcare workers. In this study, we evaluated occupational exposure to cytostatic drugs in hospital workers from the University Hospital in Tlemcen, Algeria. Monitoring was performed by collecting wipe samples from surfaces, objects, personal protective equipment (gloves and masks) and from the skin of employees at an Algerian university hospital. Wipe samples were analyzed with ultra-performance liquid chromatography coupled to a mass spectrometer. Concentrations ranged from below the limit of quantification up to 208.85, 23.45, 10.49, and 22.22 ng/cm^2^ for cyclophosphamide, ifosfamide, methotrexate and 5-fluorouracil, respectively. The highest values were observed in the oncology department. Nowadays, there are still no safe threshold limit values for occupational exposure to cytostatic agents. Therefore, contamination levels should be kept as low as reasonably achievable. Yet, healthcare workers in this hospital are still exposed to cytostatic agents, despite the numerous guidelines, and recommendations. Consequently, actions should be taken to reduce the presence of harmful agents in the work environment.

## Introduction

Cytostatic drugs or antineoplastic drugs are designed to damage and kill cancer cells. They are frequently used in cancer patients as chemotherapy. During preparation and administration of these harmful agents, healthcare workers can also be exposed. Exposure can occur via contact with contaminated work surfaces, equipment and patients' excreta, by manipulation of solutions containing cytostatic agents, by cleaning or by inhaling particles resulting from these actions. Dermal exposure can in turn lead to ingestion by hand-to-mouth contact (1–4). Healthcare workers are often exposed multiple times a week for several years. Since in this case cytostatic drugs only affect healthy cells, healthcare workers could potentially encounter several side effects. Mutagenic, developmental, reproductive effects and cancer were reported in the NIOSH (National Institute for Occupational Safety and Health) alert from 2004 (5). Up till now, there are still no official exposure limits for cytostatic drugs. Some research groups formulated their own recommendations in terms of safe exposure values, e.g., Sessink (6) recommends values below 0.1 ng/cm^2^ for cyclophosphamide. Yet, the exposure and thus the level of environmental contamination should be kept as low as reasonably achievable (5).

Multiple recommendations and guidelines have been published by among others, OSHA (Occupational Safety and Health Administration), ASHP (American Society of Hospital Pharmacists), NIOSH, ISOPP (International Society of Oncology Pharmacy Practitioners), and the Oncology Nursing Society (5, 7–10). These standards consist of recommendations on a wide range of subjects, including transport of cytostatic drugs, education and training of staff, (personal) protective equipment, monitoring of contamination, cleaning procedures, waste handling etc. Even though many recommendations and guidelines led to a decrease in occupational exposure, there is still no complete elimination of exposure (11–13). Several research groups demonstrated that even after introduction of these measures, cytostatic agents remain widespread on various surfaces and objects (3, 12–16). This can be due to non-compliancy with guidelines, limited resources or inadequacy of protocols (e.g., cleaning). Particularly in low- and middle-income countries, the limited resources can play an important role in exposure of healthcare workers and irregular environmental monitoring. Since engineering controls such as biosafety cabinets are costly, healthcare workers in low- and middle-income countries have to rely more on other measures to control hazard (e.g., personal protective equipment) (17). However, studies on safe handling of cytostatic drugs among healthcare workers in middle-income countries reported insufficient or complete lack of specialized training, inappropriate cleaning procedures and a high variability in awareness of potential hazards and use of personal protective equipment (18–23). In general, these studies indicate the need for a better implementation of guidelines. Therefore, environmental contamination and occupational exposure of healthcare workers should be monitored. Although many studies have investigated environmental exposure, not much research has been conducted using an integrative approach for different types of samples to map environmental contamination (24–27).

Our hypothesis was that even though many guidelines and recommendations are in place, there is still significant exposure of hospital workers to cytostatic drugs, especially in low- and middle-income countries. We evaluated surface contamination and the unintended occupational skin exposure to cytostatic drugs (cyclophosphamide, ifosfamide, methotrexate and 5-fluorouracil) in hospital workers from the University Hospital in Tlemcen, Algeria.

## Materials and Methods

### Chemicals, Reagents, and Materials

Ifosfamide (IFO) and methotrexate (MTX) standards were European Pharmacopeia Reference Standards. Cyclophosphamide (CP) and deuterated CP (CP-d4) were purchased from Santa Cruz Biotechnology, Inc. (Dallas, Texas, USA). 5-fluorouracil (5-FU), 5-fluorouracil-2-13C, 15N2 (5-FU13C15N2), deuterated MTX (MTX-d3) as well as ammonium formate (AF) were purchased from Sigma-aldrich (Saint Louis, Missouri, USA). Formic acid (FA) for LC-MS was purchased from Fluka (Honeywell International Inc., New Jersey, USA). Ammonia was purchased from Chem-Lab Analytical (Zedelgem, Belgium) and UPLC-MS grade water from Biosolve (Valkenswaard, The Netherlands). LC-MS grade methanol (MeOH) from J.T. Baker and HPLC-MS grade acetonitrile (ACN) were purchased from VWR International (Radnor, Pennsylvania, USA). A rotator, model reax 2 from Heidolph, Schwabach, Germany was used.

### Preparation of Stock Solutions

Stock solutions of CP-d4 and 5-FU13C15N2 in MeOH with a final concentration of 100 μg/mL were made. MTX-d3 was received as a 100 μg/mL solution. These stock solutions were diluted with MeOH/ACN (8/2), resulting in three stock solutions of 10 μg/mL. Calibration stock solutions for each compound with a final concentration of 1 mg/mL in MeOH were prepared. Calibration stock solutions of 10 μg/mL were made in MeOH/water (8/2).

### Analytical Procedure

Since the compounds of interest have different physicochemical properties, two separate methods were applied in order to measure the concentration of the compounds in environmental samples.

#### Cyclophosphamide, Ifosfamide, and Methotrexate

CP, IFO, and MTX were detected in the samples reconstituted in water/MeOH (9/1), based on a validated method, with some minor adaptations (28). Analysis was performed with an Acquity UPLC M-class system (Waters, Milford, Massachusetts, USA) and compounds were separated on a Luna Omega 1.6 μm C18 100 Å, 2.1 × 50 mm column (Phenomenex, Torrance, California, USA). Mobile phase A consisted of 0.1% FA in water and B of MeOH. Two separate gradient profiles were used (Supplementary Table 1). A flow rate of 0.4 mL/min and an injection volume of 10 μL were used. The outlet of the column was coupled to a Quattro Premier triple quadrupole mass spectrometer (Waters, Milford, Massachusetts, USA) with ESI ion source. The positive ion mode was used with multiple reaction monitoring. A source temperature of 120°C and a desolvation temperature of 450°C were used together with a capillary voltage of 1.50 kV. The desolvation and cone gas flow were 800 and 25 L/h, respectively. Other MS/MS parameters can be found in Supplementary Table 2.

#### 5-Fluorouracil

Analysis was performed using the method of Oriyama et al. (29), with some small modifications. An Acquity UPLC H-class PLUS system (Waters, Milford, Massachusetts, USA) was used for analysis. Samples reconstituted in ACN were injected in an Acquity UPLC BEH amide 1.7 μm, 2.1 × 50 mm column (Waters, Milford, Massachusetts, USA) for separation of 5-FU and 5-FU13C15N2. 0.01% FA in ACN was used as mobile phase A and 10 mM AF with 0.05% ammonia in water as mobile phase B. For gradient profile see Supplementary Table 1. A flow rate of 0.4 ml/min and an injection volume of 10 μL were used. A Xevo TQ-XS tandem quadrupole Mass Spectrometer (Waters, Milford, Massachusetts, USA) with a UniSpray ion source was used for detection. Negative ion mode was used in multiple reaction monitoring. An impactor voltage of 0.50 kV and desolvation temperature of 450°C were applied. A desolvation, cone and nebuliser gas flow of 600, 150 L/h, and 7.0 bar were used, respectively (Supplementary Table 2).

### Method Validation

An extraction solvent containing 20 ng/mL of internal standards CP-d4, MTX-d3 and 5-FU13C15N2 was prepared by mixing each internal standard stock solution with MeOH/ACN (8/2). The first calibration working solution with final concentrations of 1,000 ng/mL containing CP, IFO, MTX, and 5-FU was made by mixing each calibration stock solution together in MeOH/water (8/2). The second calibration working solution was obtained by diluting the first calibration working solution with MeOH/water (8/2) to result in a final concentration of 50 ng/mL. These two calibrations working solutions were used to spike TX714K low TOC Alpha Swab Series of 100% polyester (Texwipe, Kernersville, North Carolina, USA) with increasing concentrations of cytostatic drugs (0, 1, 5, 10, 30, 70, 100, 300 ng/swab). This was done in triplicate. After spiking, the swabs for calibration were each placed in separate glass vials and snapped at the notch of the handle. Ten mL of extraction solvent containing 20 ng/mL of internal standards, was added to the glass vials containing the swabs. The vials were shaken and rotated for 30 min. Subsequently, two times 4.5 mL was transferred to separate test tubes and evaporated under a nitrogen gas stream. The dry samples were then reconstituted in 900 μL MeOH/ACN (8/2), vortexed and transferred to injection vials. These vials were again dried out and half of them was reconstituted in 300 μL water/MeOH (9/1), while the other half was reconstituted in 300 μL ACN. After vortexing, the samples were injected. The limit of quantification (LOQ) was defined as the lowest concentration for which the precision was below 20% and the accuracy between 80 and 120%. The accuracy was calculated as the average of the estimated concentration divided by the nominal concentration, multiplied by 100 and the precision as the standard deviation of the estimated concentration divided by the average of the estimated concentration, multiplied by 100.

### Field Study: Evaluation of Surface Contamination and Occupational Exposure

In a university hospital in Algeria, samples were collected in 6 different departments. More specifically, surfaces, objects, personal protective equipment (PPE), and the skin of healthcare workers were sampled in the dermatology, maternity oncology, oncology, hematology, nephrology, and rehabilitation departments.

#### Sample Collection

Cytostatic drug sampling kits containing swabs, a square template with a 10 × 10 cm opening and a vial containing MeOH/water (8/2) were provided by the Laboratory for Occupational and Environmental Hygiene (LOEH, Leuven, Belgium) for surface sampling. Surface samples were collected using TX714K Low TOC Alpha Swab Series (Texwipe, Kernersville, North Carolina, USA). After the swab was immersed in MeOH/water (8/2), it was pushed against the walls of the vial and wiped across the rim to remove air and expel any excess of solvent. This is important to avoid inconsistent results. The swab was then used to wipe a surface using the provided template and according to a specific pattern. The first side of the first swab was wiped horizontally across the opening of the template, the second side was used to wipe the same area vertically. A second swab was used if the surface area was over 100 cm^2^ and when possible, the area was measured. The second swab was used to wipe the area diagonally upwards with one side and diagonally downwards with the other side. After sampling, both swabs were placed in the glass vial containing the remaining solvent. The vial was then closed and stored at −20°C until shipment to Belgium. Dermal samples and samples from objects were taken by use of the same sampling kit. PPE sampling involved wiping the front and back side of gloves and facial masks using two swabs and was performed in the Laboratory for Occupational and Environmental Hygiene (Leuven, Belgium). All samples were stored at −80°C until use.

#### Sample Extraction

Ten mL of extraction solvent was added to the glass vials containing the swabs. From this point forward, exact the same process was followed as for the calibration samples.

## Results

### Method Validation

Calibration curves were based on eight concentration levels (0, 1, 5, 10, 30, 70, 100, and 300 ng/swab). Curves for all compounds had correlation coefficients *R*^2^ exceeding 0.99. The limits of quantification (LOQs) were based on the definition mentioned in the materials and methods section and were 10 ng/sample for CP, 30 ng/sample for IFO, 5 ng/sample for MTX and 10 ng/sample for 5-FU. More detailed information on accuracy, precision and linearity is summarized in Table 1.

**Table 1:** Method validation parameters.

**Compound**	**Accuracy (%)**	**Precision (%)**	***R*^2^**	**LOQ (ng/sample)**
CP^a^	109.97	4.31	0.9959	10
IFO^a^	80.54	7.52	0.9989	30
MTX^a^	105.26	6.34	0.9968	5
5-FU^a^	95.76	4.59	0.9962	10

a *CP, cyclophosphamide; IFO, ifosfamide; MTX, methotrexate; 5-FU, 5-fluorouracil*.

### Field Study: Evaluation of Surface Contamination and Occupational Exposure

**Figure 1:**
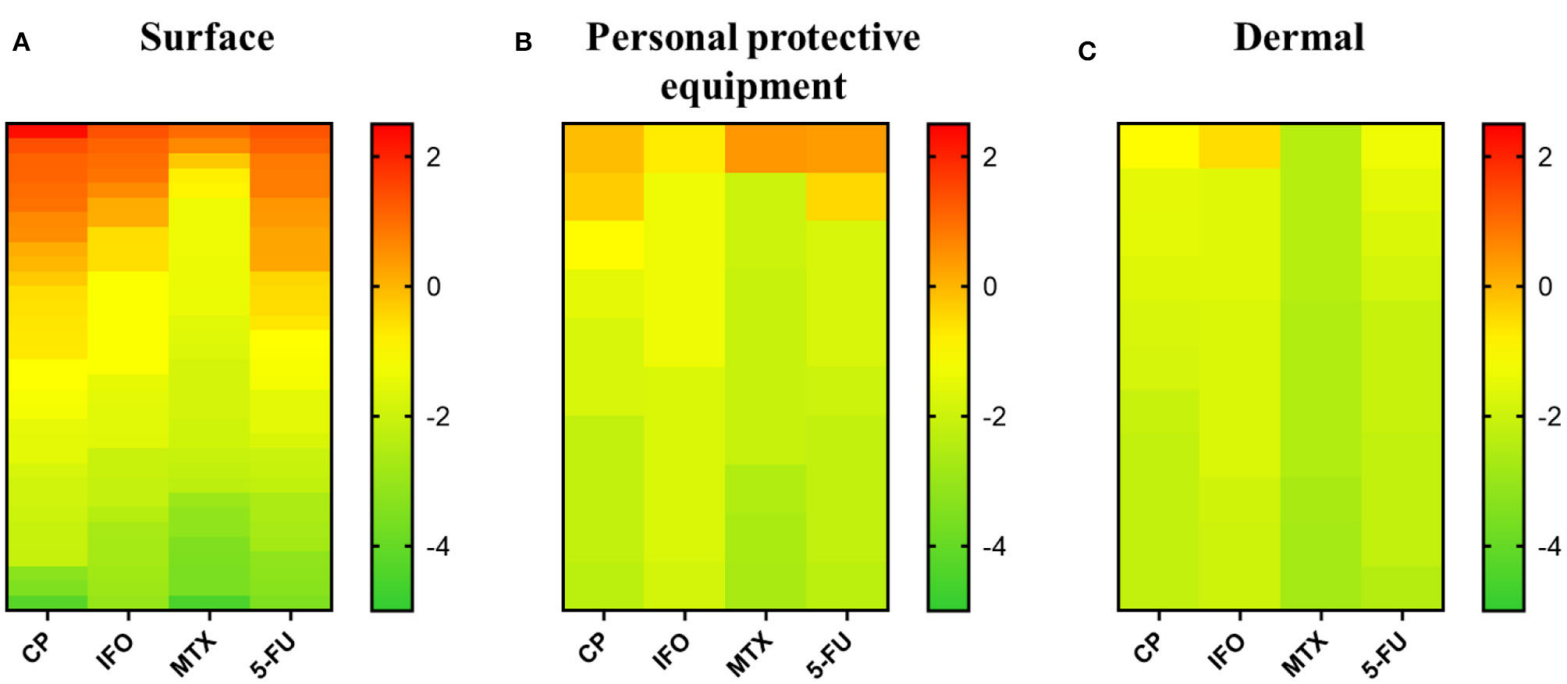
Overview of cytostatic drug concentration in surface, personal protective equipment and dermal samples from the university hospital, Algeria. All three panels give the logarithmic scale of cytostatic concentrations (ng/cm^2^) ranked from high to low for each cytostatic drug. **(A)** Cytostatic concentrations found in surface samples. **(B)** Cytostatic concentrations found in samples of personal protective equipment. **(C)** Cytostatic concentrations in dermal samples. CP, cyclophosphamide; IFO, ifosfamide; MTX, methotrexate; 5-FU, 5-fluorouracil.

In total 62 samples were collected in 6 different departments in the University Hospital. These samples included 39 surface samples, 10 samples from PPE and 13 dermal samples. Surface samples were collected from various areas and objects such as door handles, tables, hoods, a calculator, a telephone, sinks, chairs… Samples from PPE were taken from gloves and facial masks. Face, hands and arms were swabbed to collect dermal samples. In general, the highest concentrations were observed in surface samples, followed by samples from PPE, and dermal samples (Figure 1).

#### Surface Samples

In case surface areas were measured, concentrations were recalculated to ng/cm^2^ to present the results. All surface samples tested positive on one or more cytostatic compounds. More than half of the 39 analyzed samples tested positive for CP (79.5%), MTX (56.4%), and 5-FU (66.7%). IFO was above the LOQ in 30.8% of the samples. CP was not only found in the highest number of positive samples, but also in the highest measured concentration per cm^2^ (208.85 ng/cm^2^) (Figure 1A). The maximum surface contaminations for IFO, CP and 5-FU were found on a calculator in the oncology department (23.45, 208.85, and 22.22 ng/cm^2^, respectively) The highest concentration of MTX was found on a work schedule in the same department (10.49 ng/cm^2^). In the oncology department all but one sample tested positive for MTX. Also, substantial concentrations of CP, IFO, and 5-FU were measured in samples from the hood before and after preparation, the telephone, the water tap after preparation and the door handle. All surface samples in the rehabilitation department tested positive for 5-FU. IFO and MTX were detected in low concentrations on a table in the treatment room (0.00–0.01 ng/cm^2^) and a non-identified sample (253.6 ng/swab IFO, 66.4 ng/swab MTX). CP could be found on a door handle (0.22 ng/cm^2^) and the same unidentified sample (639.1 ng/swab). Only one surface sample from the nephrology department was provided. This sample, taken from the preparation table, contained a high concentration of CP (25.25 ng/cm^2^) and only a low concentration of 5-FU (0.01 ng/cm^2^). The concentrations of IFO and MTX were below the LOQ. All but one sample collected in the hematology department tested positive for CP. Low concentrations of IFO, MTX and 5-FU were observed in samples from a drawer (0.03 ng/cm^2^ IFO), fridge (0.03 ng/cm^2^ IFO, 0.01 ng/cm^2^ MTX and 5-FU), tap (0.54 ng/cm^2^ MTX, 0.07 ng/cm^2^ 5-FU), and sink (0.04 ng/swab MTX). Except for one sample from a chair (0.03 ng/cm^2^ IFO), all samples in the maternity oncology department had IFO concentrations below the LOQ. CP was observed to be above the LOQ in all surface samples. Especially, a sample from the hood after use contained a high concentration of CP (13.84 ng/cm^2^). MTX was observed in samples of the sink, tap and a non-identified sample. 5-FU was detected in quantifiable concentrations on the hood after use (1.70 ng/cm^2^) and on the same chair (0.06 ng/cm^2^), sink (0.21 and 0.02 ng/cm^2^), tap (6.07 ng/cm^2^), and non-identified sample. In the dermatology department, all surface samples tested positive for CP. IFO could only be quantified on a door handle (1.28 ng/cm^2^), MTX on a door handle (4.01 ng/cm^2^) and a preparation table (0.001 ng/cm^2^) and 5-FU on a door handle (1.71 ng/cm^2^) and in a sample of the preparation table (0.002 ng/cm^2^). More detailed information can be found in Supplementary Table 3.

#### Samples of PPE

The concentrations found on masks were recalculated to ng/cm^2^ based on the total surface area (315 cm^2^). For the gloves, a total surface area of 800 cm^2^ was used to recalculate, consistent with the surface area of hands according to the World Health Organization (30). Seven out of 10 analyzed samples contained at least one cytostatic compound. Four samples contained CP in concentrations that exceeded the LOQ, one sample for IFO, three for MTX, and three for 5-FU. The highest concentration was observed for MTX (2.66 ng/cm^2^) (Figure 1B). IFO (0.18 ng/cm^2^) was only quantifiable in gloves used for preparation by a nurse working in the oncology department. CP was quantified on gloves from a nursing aide in the nephrology department (0.50 ng/cm^2^) and gloves (0.79 ng/cm^2^) and two masks (0.03 and 0.11 ng/cm^2^) from nurses in the oncology department. Substantial concentrations of MTX (2.66 ng/cm^2^) were detected on gloves from the rehabilitation department, whereas low concentrations were seen in samples of gloves from the maternity oncology (0.01 ng/cm^2^) and dermatology (0.01 ng/cm^2^) departments. Low concentrations of 5-FU were found on the gloves of a nurse from the nephrology department (0.01 ng/cm^2^) and a mask from the oncology department (0.34 ng/cm^2^), while a higher concentration of 2.15 ng/cm^2^ was detected on the gloves used for the preparation of cytostatic drugs in the oncology department. For further information, consult Supplementary Table 3.

#### Dermal Samples

Also for the samples collected from the skin, recalculations were performed according to surface areas mentioned by the World Health Organization (30). Surface areas of 800 cm^2^ for hands, 1,200 cm^2^ for arms and 650 cm^2^ for the face were used. Eight out of 13 analyzed dermal samples tested positive for one or more cytostatic compounds. CP was found in quantifiable concentrations in 7 out of 13 samples. IFO was only present in one sample, 5-FU in five samples and MTX in none of the tested samples. The highest concentration was measured on the face of a nurse active in the oncology department (0.28 ng/cm^2^ IFO) (Figure 1C). None of the samples contained MTX concentrations above the LOQ. CP was present in only low concentrations on the face and arms of the same nurse from the oncology department (0.11 ng/cm^2^ on face and 0.03 ng/cm^2^ on arms), the faces of two nurses in the hematology department (0.02 ng/cm^2^) and body samples from the nurse and psychologist from the maternity oncology department (0.03 and 0.02 ng/cm^2^). For 5-FU low concentrations were detected in samples from the faces of nurses in the nephrology (0.03 ng/cm^2^) and oncology (0.05 ng/cm^2^) departments, arms of a nurse in the oncology department (0.03 ng/cm^2^), body samples of a psychologist (0.01 ng/cm^2^) and hands of a nurse from the rehabilitation department (0.02 ng/cm^2^). Further information is listed in Supplementary Table 3.

## Discussion

The aim of this study was to evaluate the environmental cytostatic contamination and unintended skin exposure in healthcare workers of an Algerian university hospital, by means of surface sampling. Absorption through the skin is the main route of occupational exposure for cytostatic agents (31). This implicates that surface contamination of hazardous substances possess an important exposure risk. As expected, the highest surface concentrations of cytostatic compounds were observed in the oncology department while the lowest concentrations were found in the rehabilitation and nephrology departments. This is in line with what was expected, since cytostatic drugs are most often used in the oncology department as part of cancer therapy. Out of the 33 samples with known surface area, 11 had a concentration below 0.1 ng/cm^2^ for all cytostatic drugs. These samples were collected in the rehabilitation, oncology, hematology, maternity oncology and dermatology department. In 14 samples, levels from 0.1 up to 10 ng/cm^2^ were detected. Low surface contamination indicates that healthcare workers were handling cytostatic drugs with care and that the cleaning procedures were adequately performed. Nonetheless, this can also be due to less frequent use of cytostatics in these departments. Low levels can still lead to exposure of healthcare workers when touched with bare hands (e.g., door handle). Even though most of the samples had concentrations below 10 ng/cm^2^, 8 out of 33 samples contained extremely high concentrations of cytostatic drugs. Six of these samples were from the oncology department. In this department, samples from the hood contained 10.21 ng/cm^2^ CP (before preparation) and 14.06 ng/cm^2^ 5-FU (after preparation). As cytostatic drugs are most often handled inside biosafety cabinets, these high concentrations were expected. The high concentrations before use of the hood do indicate poor cleaning procedures. On the calculator, phone, work schedule, and door handle of the oncology department, concentrations exceeding 10 ng/cm^2^ were found. The surface samples from the calculator contained even higher concentrations of several cytostatic compounds (208.85 ng/cm^2^ CP, 23.45 ng/cm^2^ IFO, 22.22 ng/cm^2^ 5-FU) than observed on the hood after preparation of these compounds. This is probably due to spillage or secondary transfer by contact in combination with inadequate cleaning. Contamination of the telephone and door handle can form a risk for the hospital personnel, since these are often handled with bare hands. In the nephrology and maternity oncology department very high concentrations of CP were observed in samples from a preparation table (25.25 ng/cm^2^) and the hood after preparation (13.84 ng/cm^2^). Such high concentrations only occur after spilling and the lack of adequate cleaning, while low surface concentrations are more an indication of careless working. High surface contamination results in a high risk for personnel to get exposed, with even the possibility to transfer this contamination to third parties. If we compare our range of surface CP contamination (< LOQ−208.85 ng/cm^2^), with the literature, which reported concentrations from < LOQ to 14 ng/cm^2^, substantially higher contamination was observed in our study (12, 25, 27, 32–35). Müller-Ramírez et al. (14) reported values ranging from 0.3 to 168.9 ng/cm^2^, which is more in line with our results, while only one study reported ranges exceeding ours with contamination up to 21,300 ng/cm^2^ (36). This concentration was found on a phone in a room next to the preparation room and was probably caused by touching the phone with contaminated gloves and inadequate cleaning. For IFO we observed concentrations ranging from < LOQ up to 23.45 ng/cm^2^, which is in line with concentrations (0.08–15.7 ng/cm^2^) reported by Müller-Ramírez et al. (14). Other studies reported substantially lower contamination (12, 25, 33, 35), or considerably higher contamination (< LOQ−95 ng/cm^2^) (32, 37). Hedmer and Wohlfart (32) described one really high concentration of 95 ng/cm^2^ on the floor of a patient lavatory, which is probably coming from the patients' excreta. In our study MTX concentrations ranged from < LOQ to 10.49 ng/cm^2^. Also for MTX surface contamination, low to very high concentrations are reported in literature, ranging from 0.515 to 51 ng/cm^2^ (12, 33, 37). Finally, in our study surface 5-FU concentrations ranged from below the LOQ to 22.22 ng/cm^2^, which is similar to Kiffmeyer et al. (37). Lower ranges were found by Koller et al. (27) and Kopp et al. (33) from < 0.007 up to 14.56 ng/cm^2^. Schierl et al. (38) and Viegas et al. (36) reported higher ranges (< LOQ−253.33 ng/cm^2^). Interpretation of the level of contamination is difficult, since there are no official threshold limit values. Yet, several research groups have already described this problem in different ways and have proposed own “in-house” thresholds. Schierl et al. (38) used the median and 75th percentile of all observed concentrations in surface samples to define two threshold values, namely concentrations below the median indicate good working practices, while values above the 75th percentile indicate the need for optimization of the handling procedures. For 5-FU these thresholds are 0.03 ng/cm^2^ for the lower limit and 0.005 ng/cm^2^ for the upper limit. Likewise, other research groups used the 90th percentile as guidance value. Kiffmeyer et al. (37) established a guidance value of 0.1 ng/cm^2^, independent of the substance measured. This value was based on the highest concentration of all the compounds that were quantified. Different guidance values for different sampling areas and rooms where cytostatic drugs are handled were proposed by Hedmer and Wohlfart (32). Sottani et al. (39) used the 90th percentile to propose guidance values for CP (3.6 ng/cm^2^), 5-FU (1.0 ng/cm^2^), gemcitabine (0.9 ng/cm^2^), and platinum (0.5 ng/cm^2^). Furthermore, Sessink (6) coupled guidance values to actions to be taken to ensure a safe environment. CP is commonly used in chemotherapy. It can permeate the skin easily and is extremely toxic. Therefore, determination of guidance values was based on the 90th and 99th percentile of CP concentrations in wipe samples. The 90th and 99th percentile correspond to 0.1 and 10 ng/cm^2^, respectively. Surface concentrations below 0.1 ng/cm^2^ are presumed to be safe, while levels above 10 ng/cm^2^ are considered to be unacceptable. Depending on the concentration found, different actions should be performed. For concentrations lower than 0.1 ng/cm^2^, the environment should be monitored once a year and evaluated after 4 years. Surfaces containing 0.1–10 ng/cm^2^ require risk estimation, monitoring within 3–6 months and action taking if necessary. For concentrations exceeding 10 ng/cm^2^, taking action and follow-up of these improvements is highly recommended. Applying these thresholds to our own data, we observed that 15 out of 33 surface samples had concentrations between 0.1 and 10 ng/cm^2^ for at least one compound, whereas 8 contained more than 10 ng/cm^2^ of one or more cytostatic drugs. In other words, ~70% of the surface samples contained levels exceeding the safe level according to Sessink (6). This points at an urgent need for action. Since it can be difficult to reduce exposure by use of costly engineering controls in middle-income countries, such as Algeria, the focus should be on administrative controls as well as personal protective equipment. Administrative controls include education and training, safe handling policies and medical surveillance. Education and training can raise awareness of hospital personnel on the potential hazards of working with cytostatic agents. Previous research in low- and middle-income countries showed that this could be a reason for non-compliancy with safety guidelines (18–23). Some extremely high levels of cytostatics were probably caused by spilling and remained on the surfaces by inadequate cleaning or cleaning protocols. This source of exposure can be reduced by adequate safe handling policies and procedures and by provision of proper equipment (e.g., biosafety cabinets, PPE, closed-system drug transfer device). Our results suggest that the current cleaning procedures should be checked by regularly performing measurements before and after cleaning. If the existing cleaning protocols then seem inadequate, changes need to be made to the protocol, and monitoring should show improvement. Since there was a lack of functioning biosafety cabinets and PPE, urgent investments are necessary. This should include spill kits and PPE in areas where harmful agents are handled, along with preferably closed-system drug transfer devices. Sessink et al. (40) found a substantially lower environmental contamination in hospitals after using the closed-system drug transfer devices compared to using standard drug preparation techniques. Finally, in this case, medical surveillance and environmental surveillance is highly recommended due to the high concentrations found on several surfaces and objects.

Next to surface sampling, we collected samples from PPE. Here, we also found the highest concentrations of CP, IFO, and 5-FU in the oncology department. MTX was found in the highest concentration in the rehabilitation department. In the other departments, low concentrations were measured. In literature, CP concentrations up to 0.68 ng/cm^2^ on gloves were reported, which is in line with our findings (< LOQ−0.79 ng/cm^2^) (24, 26, 27, 41). For IFO concentrations ranging between 0.14 and 2.26 ng/cm^2^ are described, whereas our results (0.18 ng/cm^2^) are at the lower end of this range (24, 26). On the other hand, we found MTX concentrations up to 2.66 ng/cm^2^, while another study showed much lower concentrations on PPE (below 0.1 ng/cm^2^) (24). For 5-FU, we found a maximum concentration of 2.15 ng/cm^2^ on gloves. While some authors reported values well below this concentration (0.018–0.33 ng/cm^2^), others reported concentrations high above these values (449.3 and 11.4 ng/cm^2^) (26, 27, 36, 41). In total, 10 samples were analyzed from PPE (masks and gloves). Six out of these samples had concentrations below 0.1 ng/cm^2^ for all compounds of interest, whereas the remaining 4 samples all had concentrations below 10 ng/cm^2^. Even though there were no extremely high concentrations detected, 7 out of 10 samples contained quantifiable concentrations of at least one cytostatic compound. This can be caused by spilling, transfer from contaminated surfaces and objects (e.g., vials) to gloves and masks. The most important safety measures that should be taken in terms of PPE are first of all the unconditional use of PPE and the regularly changing of gloves, mask and lab gown, especially after spills.

In total, 13 dermal samples with known surface area were analyzed. None of these samples contained high concentrations of cytostatic drugs. Few studies have been measuring dermal exposure to cytostatic drugs by use of the wiping technique (42–45). Hon et al. (42, 43) reported CP concentrations up to 0.028 ng/cm^2^ on the skin, while in this study higher concentrations up to 0.11 ng/cm^2^ were observed. Hon et al. (42) also reported low dermal MTX contamination ranging from < LOQ to 0.0003 ng/cm^2^. Although there was substantial MTX in the surface samples, we found no MTX on the skin (LOQ < 5 ng/sample). Five out of 13 samples had concentrations below the LOQ for all 5 compounds, 7 had concentrations between 0.01 and 0.03 ng/cm^2^ and only 1 had a concentration exceeding 0.1 ng/cm^2^. This exposure can be caused by touching the face with contaminated gloves. Therefore, it is important to wear PPE and to avoid contact with bare skin. Bos et al. (46) proposed a dermal occupational exposure limit of 4 ng/cm^2^ for CP. There were some limitations in our study. A first one is that not all vials were packed well for shipment, resulting in partial loss of some samples and turning some identification labels unreadable. Also, not all surface areas were measured because of their complex shape. Due to the unreadable labels and missing areas of some surfaces, the results of 6 samples could not be compared to other samples (in ng/cm^2^) or interpreted. We also only had one surface sample from the nephrology department, which makes it difficult to get a good view of this department. For PPE and dermal samples we only had a limited number of samples. Therefore, it is difficult to estimate the actual exposure of the health care workers in these departments. We also have to take into account that the sampled area is not necessarily representative for the total surface of the object (e.g., hood, table).

In the future, this environmental monitoring needs to be complemented by biological monitoring. A combination of both approaches will give a more profound insight in the actual occupational exposure of healthcare workers.

## Conclusion

Despite the numerous guidelines and recommendations, there is still a significant exposure of healthcare workers to cytostatic drugs in the hospital examined. This study offers a first perspective on the occupational exposure in an Algerian healthcare setting and shows that significant concentrations of hazardous compounds are present on a broad range of surfaces and objects in different departments of the hospital. These results can be interpreted as a call for action. Regular environmental and biological monitoring can give a clear idea about the corrective actions to be taken in the future and will further enable follow-up of improvements.

## Data Availability Statement

All datasets presented in this study are included in the article/Supplementary Material.

## Author Contributions

EV, MC, JV, MZ, TA, RL, KP, R-CD, and LG: conception and design. EV and MC: analysis. EV, MC, LG, R-CD, KP, and JV: interpretation. EV and MC: drafting manuscript. LG, R-CD, KP, JV, RL, MZ, and TA: proof reading. All authors contributed to the article and approved the submitted version.

## Conflict of Interest

LG was employed by the company Idewe. The remaining authors declare that the research was conducted in the absence of any commercial or financial relationships that could be construed as a potential conflict of interest.

## References

[1] Sessink PJM Sewell G Vandenbroucke J Preventing Occupational Exposure to Cytotoxic and Other Hazardous Drugs European Policy Recommendations. (2016). Available online at: http://www.europeanbiosafetynetwork.eu/wp-content/uploads/2016/05/Exposure-to-Cytotoxic-Drugs_Recommendation_DINA4_10-03-16.pdf (accessed October 22, 2019).

[2] Ratner PA Spinelli JJ Beking K Lorenzi M Chow Y Teschke K. Cancer incidence and adverse pregnancy outcome in registered nurses potentially exposed to antineoplastic drugs. BMC Nurs. (2010) 9:15. 10.1186/1472-6955-9-15 20846432 PMC2949748

[3] Fabrizi G Fioretti M Mainero Rocca L. Dispersive solid-phase extraction procedure coupled to UPLC-ESI-MS/MS analysis for the simultaneous determination of thirteen cytotoxic drugs in human urine. Biomed Chromatogr. (2016) 30:1297–308. 10.1002/bmc.3684 26762960

[4] Centers for Disease Control and Prevention (a) Antineoplastic Agents - Occupational Hazards in Hospitals. (2004). Available online at: https://www.cdc.gov/niosh/docs/2004-102/default.html (accessed October 22, 2019).

[5] Centers for Disease Control and Prevention (b) NIOSH Alert - Preventing Occupational Exposures to Antineoplastic and Other Hazardous Drugs in Health Care Settings. (2004). Available online at: https://www.cdc.gov/niosh/docs/2004-165/default.html (accessed October 22, 2019).

[6] Sessink PJM Environmental contamination with cytostatic drugs: past, present and future. Saf Consid Oncol Pharm. Special edition, Fall. (2011). Available online at: https://www.semanticscholar.org/paper/Environmental-contamination-with-cytostatic-drugs%3A-Sessink/86524d606dbd8e116ca2c26b1ec70d3f81cfe272 (accessed October 22, 2019).

[7] Connor T McLauchlan R Vandenbroucke J. ISOPP safe handling of cytotoxics. J Oncol Pharm Pract. (2007) 13:1–81. 10.1177/1078155207082350 17933809

[8] Polovich M. Safe handling of hazardous drugs. Online J Issues Nurs. (2004) 9:1–18. 15482092

[9] American Society of Health-System Pharmacists ASHP guidelines on handling hazardous drugs. Am J Health Syst Pharm. (2006) 63:1172–91. 10.2146/ajhp050529

[10] Occupational and Safety and Health Administration Controlling Occupational Exposure to Hazardous Drugs. (2016). Available online at: https://goo.gl/FQZ9Ta (accesed June 25, 2020).

[11] Fransman W Peelens S Hilhorst S Roeleveld N Heederik D Kromhout H. A pooled analysis to study trends in exposure to antineoplastic drugs among nurses. Ann Occup Hyg. (2007) 51:231–9. 10.1093/annhyg/mel081 17337460

[12] Merger D Tanguay C Langlois É Lefebvre M Bussières JF. Multicenter study of environmental contamination with antineoplastic drugs in 33 canadian hospitals. Int Arch Occup Environ Health. (2014) 87:307–13. 10.1007/s00420-013-0862-0 23471647

[13] Böhlandt A Schierl R Benefits of wipe sampling: evaluation of long-term 5-fluorouracil and platinum monitoring data. Pharm Technol Hosp Pharm. (2016) 1:139–50. 10.1515/pthp-2016-0010

[14] Müller-Ramírez C Squibb K McDiarmid M. Measuring extent of surface contamination produced by the handling of antineoplastic drugs in low- to middle-income country oncology health care settings. Arch Environ Occup Health. (2017) 72:289–98. 10.1080/19338244.2016.1222346 27603111

[15] Roland C Caron N Bussières JF. Multicenter study of environmental contamination with cyclophosphamide, ifosfamide, and methotrexate in 66 canadian hospitals: a 2016 follow-up study. J Occup Environ Hyg. (2017) 14:650–8. 10.1080/15459624.2017.1316389 28574754

[16] Mason HJ Blair S Sams C Jones K Garfitt SJ Cuschieri MJ. Exposure to antineoplastic drugs in two UK hospital pharmacy units. Ann Occup Hyg. (2005) 49:603–10. 10.1093/annhyg/mei023 15964878

[17] Pan American Health Organization Safe Handling of Hazardous Chemotherapy Drugs in Limited-Resource Settings. (2012). Available online at: https://www.paho.org/hq/dmdocuments/2014/safe-handling-chemotherapy-drugs.pdf (accessed June 25, 2020).

[18] Shahrasbi AA Afshar M Shokraneh F Monji F Noroozi M Ebrahimi-Khojin M. Risks to health professionals from hazardous drugs in Iran: a pilot study of understanding of healthcare team to occupational exposure to cytotoxics. EXCLI J. (2014) 13:491–501. 10.17877/DE290R-16003 26417276 PMC4464082

[19] Zayed HA Saied SM El-Sallamy RM Shehata WM Knowledge, attitudes and practices of safe handling of cytotoxic drugs among oncology nurses in Tanta University Hospitals. Egypt J Occup Med. (2019) 43:75–92. 10.21608/ejom.2019.25119

[20] Nwagbo S Ilesanmi R Ohaeri B Oluwatosin A Knowledge of chemotherapy and occupational safety measures among nurses in oncology units. J Clin Sci. (2017) 14:131–7. 10.4103/jcls.jcls_88_16

[21] Alehashem M Baniasadi S. Important exposure controls for protection against antineoplastic agents: highlights for oncology health care workers. Work. (2018) 59:165–72. 10.3233/WOR-172656 29439374

[22] Elshaer NS. Adverse health effects among nurses and clinical pharmacists handling antineoplastic drugs: adherence to exposure control methods. J Egypt Public Health Assoc. (2017) 92:144–55. 10.21608/epx.2017.16392 30341993

[23] Hosen MS Hasan M Saiful Islam M Raseduzzaman MM Tanvirul Islam M Tazbiul Islam M Evaluation of knowledge and practice of handling chemotherapy agents by nurses: a multi-centre studies in Bangladesh. Int J Community Med Public Heal. (2019) 6:4175–80. 10.18203/2394-6040.ijcmph20194471

[24] Ziegler E Mason HJ Baxter PJ. Occupational exposure to cytotoxic drugs in two UK oncology wards. Occup Environ Med. (2002) 59:608–12. 10.1136/oem.59.9.608 12205233 PMC1740366

[25] Nussbaumer S Geiser L Sadeghipour F Hochstrasser D Bonnabry P Veuthey JL. Wipe sampling procedure coupled to LC-MS/MS analysis for the simultaneous determination of 10 cytotoxic drugs on different surfaces. Anal Bioanal Chem. (2012) 402:2499–509. 10.1007/s00216-011-5157-2 21701850

[26] Simon N Vasseur M Pinturaud M Soichot M Richeval C Humbert L. Effectiveness of a closed-system transfer device in reducing surface contamination in a new antineoplastic drug-compounding unit: a prospective, controlled, parallel study. PLoS ONE. (2016) 11:e015952. 10.1371/journal.pone.0159052 27391697 PMC4938267

[27] Koller M Böhlandt A Haberl C Nowak D Schierl R. Environmental and biological monitoring on an oncology ward during a complete working week. Toxicol Lett. (2018) 298:158–63. 10.1016/j.toxlet.2018.05.002 29738807

[28] Izzo V Charlier B Bloise E Pingeon M Romano M Finelli A. A UHPLC–MS/MS-based method for the simultaneous monitoring of eight antiblastic drugs in plasma and urine of exposed healthcare workers. J Pharm Biomed Anal. (2018) 154:245–51. 10.1016/j.jpba.2018.03.024 29558725

[29] Oriyama T Yamamoto T Yanagihara Y Nara K Abe T Nakajima K. Evaluation of the permeation of antineoplastic agents through medical gloves of varying materials and thickness and with varying surface treatments. J Pharm Heal Care Sci. (2017) 3:1–8. 10.1186/s40780-017-0082-y 28469932 PMC5412027

[30] World Health Organization IPCS. Environmental health criteria 242. Dermal exposure. IOMC. Inter-Organization Programme for the Sound Management of Chemcials. (2014). Available online at: https://www.who.int/ipcs/publications/ehc/ehc_242.pdf (accessed October 14, 2019).

[31] Connor TH Zock MD Snow AH. Surface wipe sampling for antineoplastic (chemotherapy) and other hazardous drug residue in healthcare settings: methodology and recommendations. J Occup Environ Hyg. (2016) 13:658–67. 10.1080/15459624.2016.1165912 27019141 PMC5138855

[32] Hedmer M Wohlfart G. Hygienic guidance values for wipe sampling of antineoplastic drugs in Swedish hospitals. J Environ Monit. (2012) 14:1968–75. 10.1039/c2em10704j 22692549

[33] Kopp B Schierl R Nowak D. Evaluation of working practices and surface contamination with antineoplastic drugs in outpatient oncology health care settings. Int Arch Occup Environ Health. (2013) 86:47–55. 10.1007/s00420-012-0742-z 22311009

[34] Odraska P Dolezalova L Kuta J Oravec M Piler P Synek S. Association of surface contamination by antineoplastic drugs with different working conditions in hospital pharmacies. Arch Environ Occup Health. (2014) 69:148–58. 10.1080/19338244.2013.763757 24325745

[35] Hedmer M Tinnerberg H Axmon A Jönsson BAG. Environmental and biological monitoring of antineoplastic drugs in four workplaces in a Swedish hospital. Int Arch Occup Environ Health. (2008) 81:899–911. 10.1007/s00420-007-0284-y 18066576

[36] Viegas S Pádua M Veiga AC Carolino E Gomes M. Antineoplastic drugs contamination of workplace surfaces in two Portuguese hospitals. Environ Monit Assess. (2014) 186:7807–18. 10.1007/s10661-014-3969-1 25096642

[37] Kiffmeyer TK Tuerk J Hahn M Stuetzer H Hadtstein C Heinemann A. Application and assessment of a regular environmental monitoring of the antineoplastic drug contamination level in pharmacies - the MEWIP project. Ann Occup Hyg. (2012) 57:444–55. 10.1093/annhyg/mes081 23125441

[38] Schierl R Böhlandt A Nowak D. Guidance values for surface monitoring of antineoplastic drugs in german pharmacies. Ann Occup Hyg. (2009) 53:703–11. 10.1093/annhyg/mep050 19620232

[39] Sottani C Grignani E Oddone E Dezza B Negri S Villani S. Monitoring surface contamination by antineoplastic drugs in Italian hospitals: performance-based hygienic guidance values (HGVs) Project. Ann Work Expo Heal. (2017) 61:994–1002. 10.1093/annweh/wxx065 29028251

[40] Sessink PJM Connor TH Jorgenson JA Tyler TG. Reduction in surface contamination with antineoplastic drugs in 22 hospital pharmacies in the US following implementation of a closed-system drug transfer device. J Oncol Pharm Pract. (2011) 17:39–48. 10.1177/1078155210361431 20156932 PMC4627487

[41] Crauste-Manciet S Sessink PJM Ferrari S Jomier JY Brossard D. Environmental contamination with cytotoxic drugs in healthcare using positive air pressure isolators. Ann Occup Hyg. (2005) 49:619–28. 10.1093/annhyg/mei045 16126757

[42] Hon CY Astrakianakis G Danyluk Q Chu W. Pilot evaluation of dermal contamination by antineoplastic drugs among hospital pharmacy personnel. Can J Hosp Pharm. (2011) 64:327–32. 10.4212/cjhp.v64i5.1067 22479084 PMC3203824

[43] Hon CY Teschke K Shen H Demers PA Venners S. Antineoplastic drug contamination in the urine of Canadian healthcare workers. Int Arch Occup Environ Health. (2015) 88:933–41. 10.1007/s00420-015-1026-1 25626912

[44] Fransman W Vermeulen R Kromhout H. Dermal exposure to cyclophosphamide in hospitals during preparation, nursing and cleaning activities. Int Arch Occup Environ Health. (2005) 78:403–12. 10.1007/s00420-004-0595-1 15887018

[45] Hon CY Teschke K Demers PA Venners S. Antineoplastic drug contamination on the hands of employees working throughout the hospital medication system. Ann Occup Hyg. (2014) 58:761–70. 10.1093/annhyg/meu019 24644303

[46] Bos PM Brouwer DH Stevenson H Boogaard PJ de Kort WL van Hemmen JJ. Proposal for the assessment of quantitative dermal exposure limits in occupational environments: part 1. Development of a concept to derive a quantitative dermal occupational exposure limit. Occup Environ Med. (1998) 55:795–804. 10.1136/oem.55.12.795 9924440 PMC1757540

