# Supplementary material for: Environmental Contamination and Occupational Exposure of Algerian Hospital Workers
Source: Front Public Health. 2020 Aug 5;8:374. doi: 10.3389/fpubh.2020.00374 (PMC7419462; doi:10.3389/fpubh.2020.00374)
Supplement: Supplementary file 1 [file Data_Sheet_1.docx]

Supplementary Material

Supplementary table 1: Liquid chromatography gradient profiles

| **CP and IFO**^a^ | | |
| --- | --- | --- |
| **Time (min)** | **% A (v/v)**^b^ | **% B (v/v)**^b^ |
| 0.00 | 90 | 10 |
| 2.50 | 20 | 80 |
| 3.00 | 20 | 80 |
| 3.50 | 90 | 10 |
| 4.00 | 90 | 10 |
| **MTX**^a^ | | |
| **Time (min)** | **% A (v/v)**^b^ | **% B (v/v)**^b^ |
| 0.00 | 90 | 10 |
| 1.50 | 90 | 10 |
| 1.80 | 20 | 80 |
| 3.00 | 20 | 80 |
| 3.50 | 90 | 10 |
| 4.00 | 90 | 10 |
| **5-FU**^a^ | | |
| **Time (min)** | **% A (v/v)**^c^ | **% B (v/v)**^c^ |
| 0.00 | 95 | 5 |
| 0.40 | 95 | 5 |
| 1.00 | 50 | 50 |
| 2.00 | 50 | 50 |
| 2.10 | 95 | 5 |
| 3.00 | 95 | 5 |

^a^CP = cyclophosphamide, IFO = ifosfamide, MTX = methotrexate, 5-FU = 5-fluorouracil
^b^A = 0.1 % formic acid in water, B = methanol
^c^A = 0.01 % formic adic in acetonitrile, B = 10 mM ammonium formate with 0.05 % ammonia in water

**Supplementary table 2: MS/MS parameters**

| **Compound** | **Cone voltage**  **(V)** ^a^ | **Parent**  **(m/z)** ^a^ | **Daughter**  **(m/z)** ^a^ | **Collision energy (eV)** ^a^ |
| --- | --- | --- | --- | --- |
| CP^a^ | 33 | 260 | 77.9 | 32 |
|  |  |  | 105.8 | 18 |
|  |  |  | 120 | 24 |
|  |  |  | 140.9 | 22 |
|  |  |  | 233.3 | 16 |
| IFO^a^ | 33 | 261 | 78 | 22 |
|  |  |  | 92 | 25 |
|  |  |  | 153.8 | 22 |
|  |  |  | 181.8 | 18 |
|  |  |  | 232.8 | 16 |
| MTX^a^ | 28 | 455.3 | 174.8  307.8 | 35  20 |
| CP-d4^a^ | 31 | 264.9 | 105.9 | 18 |
|  |  |  | 123.9 | 24 |
|  |  |  | 139.9 | 22 |
|  |  |  | 234.8 | 18 |
| MTX-d3^a^ | 28 | 458.3 | 177.8  310.8 | 35  20 |
| 5-FU^a^ | 14 | 128.92 | 85.28  128.58 | 16  14 |
| 5-FU13C15N2^a^ | 18  14 | 131.96 | 87.31  131.58 | 18  14 |

^a^V = volt, m/z = mass-to-charge ratio, eV = electron volt, CP = cyclophosphamide, IFO = ifosfamide, MTX = methotrexate, CP-d4 = deuterated cyclophosphamide, MTX-d3 = deuterated methotrexate, 5-FU = 5-fluorouracil, 5-FU13C15N2 = 5-fluorouracil-2-^13^C,^15^N_2_

**Supplementary table 3: Cytostatic drug concentration in surface, personal protective equipment and dermal samples from the university hospital of Tlemcen, Algeria**

| **SURFACE SAMPLES** | | | | | | |
| --- | --- | --- | --- | --- | --- | --- |
| **Department** | **Sample** | **Concentration** | | | | |
|  |  | **CP**^a^ | **IFO**^a^ | **MTX**^a^ | **5-FU**^a^ | Ng/  cm² |
| Rehabilitation | Door handle | 0.22 | < LOQ | < LOQ | 2.46 |  |
| Rehabilitation | Table in treatment room | < LOQ | 0.00 | 0.01 | 0.00 |  |
| Rehabilitation | Handle of preparation cart | < LOQ | < LOQ | < LOQ | 0.29 |  |
| Rehabilitation | Tap treatment room | < LOQ | < LOQ | < LOQ | 0.10 |  |
| Rehabilitation | Alcohol bottle | < LOQ | < LOQ | < LOQ | 1.59 |  |
| Rehabilitation | Not identified | 639.10 | 253.62 | 66.40 | 998.81 | Ng/  sample |
| Rehabilitation | Not identified | < LOQ | < LOQ | < LOQ | 31.86 |  |
| Nephrology | Preparation table | 25.25 | < LOQ | < LOQ | 0.01 | Ng/  cm² |
| Oncology | Hood (interior, before preparation) | 10.21 | 7.85 | 0.03 | 2.75 |  |
| Oncology | Surface samples from hood (after preparation) | 3.99 | 3.73 | 0.02 | 14.06 |  |
| Oncology | Calculator (after preparation) | 208.85 | 23.45 | 0.02 | 22.22 |  |
| Oncology | Telephone (after preparation) | 13.03 | 10.15 | 0.04 | 6.06 |  |
| Oncology | Tap (after preparation) | 0.09 | < LOQ | 0.13 | 0.29 |  |
| Oncology | Tap (after preparation) | 0.28 | 1.36 | 0.04 | 0.34 |  |
| Oncology | Door handle (after preparation) | 3.42 | 13.19 | < LOQ | 6.57 |  |
| Oncology | Closet treatment room | < LOQ | < LOQ | 0.00 | < LOQ |  |
| Oncology | Work schedule | < LOQ | < LOQ | 10.49 | < LOQ |  |
| Oncology | Not identified | 71.30 | < LOQ | 110.63 | < LOQ | Ng/  sample |
| Haematology | Drawer | 0.24 | 0.03 | 0.00 | < LOQ | Ng/  cm² |
| Haematology | Hood 1 (exterior) | 0.03 | < LOQ | < LOQ | < LOQ |  |
| Haematology | Fridge | 0.01 | 0.03 | 0.01 | 0.01 |  |
| Haematology | Tap | < LOQ | < LOQ | 0.54 | 0.07 |  |
| Haematology | Sink | 0.01 | < LOQ | 0.04 | < LOQ |  |
| Haematology | Not identified | 136.30 | < LOQ | 199.96 | < LOQ | Ng/  sample |
| Haematology | Not identified | 10.66 | < LOQ | < LOQ | 96.23 |  |
| Maternity oncology | Chair at moment of maternity activity | 0.06 | 0.03 | < LOQ | 0.06 | Ng/  cm² |
| Maternity oncology | Chair after maternity activity | 0.01 | < LOQ | < LOQ | < LOQ |  |
| Maternity oncology | Sink at moment of maternity activity | 0.20 | < LOQ | 0.16 | 0.21 |  |
| Maternity oncology | Sink after maternity activity | 0.02 | < LOQ | 0.01 | 0.02 |  |
| Maternity oncology | Tap after maternity activity | 0.07 | < LOQ | < LOQ | < LOQ |  |
| Maternity oncology | Tap | 0.20 | < LOQ | 0.04 | 6.07 |  |
| Maternity oncology | Hood after activity | 13.84 | < LOQ | < LOQ | 1.70 |  |
| Maternity oncology | Soap dispenser at the moment of maternity activity | 1.26 | < LOQ | < LOQ | < LOQ |  |
| Maternity oncology | Soap dispenser after maternity activity | 0.96 | < LOQ | < LOQ | < LOQ |  |
| Maternity oncology | Not identified | 61596.19 | < LOQ | 9.17 | 4405.14 | Ng/  sample |
| Dermatology | Door handle 1 | 8.61 | 1.28 | < LOQ | 1.71 | Ng/  cm² |
| Dermatology | Door handle | 0.52 | < LOQ | 4.01 | < LOQ |  |
| Dermatology | Preparation table in treatment room 2 | 0.00 | < LOQ | 0.00 | < LOQ |  |
| Dermatology | Preparation table | 0.01 | < LOQ | < LOQ | 0.00 |  |
| **PERSONAL PROTECTIVE EQUIPMENT** | | | | | | |
| **Department** | **Sample** | **Concentration** | | | | |
|  |  | **CP**^a^ | **IFO**^a^ | **MTX**^a^ | **5-FU**^a^ | Ng/  cm² |
| Rehabilitation | Gloves | < LOQ | < LOQ | 2.66 | < LOQ |  |
| Nephrology | Gloves nursing aide | 0.50 | < LOQ | < LOQ | 0.01 |  |
| Nephrology | Mask nursing aide | < LOQ | < LOQ | < LOQ | < LOQ |  |
| Oncology | Gloves from nurse (used for preparation) | 0.79 | 0.18 | < LOQ | 2.15 |  |
| Oncology | Mask from nurse (used for preparation) | 0.03 | < LOQ | < LOQ | < LOQ |  |
| Oncology | Mask | 0.11 | < LOQ | < LOQ | 0.34 |  |
| Haematology | Gloves and mask | < LOQ | < LOQ | < LOQ | < LOQ |  |
| Maternity oncology | Mask after preparation | < LOQ | < LOQ | < LOQ | < LOQ |  |
| Maternity oncology | Gloves after preparation | < LOQ | < LOQ | 0.01 | < LOQ |  |
| Dermatology | Gloves from preparation and administration | < LOQ | < LOQ | 0.01 | < LOQ |  |
| **DERMAL SAMPLES** | | | | | | |
| **Department** | **Sample** | **Concentration** | | | | |
|  |  | **CP**^a^ | **IFO**^a^ | **MTX**^a^ | **5-FU**^a^ | Ng/  cm² |
| Rehabilitation | Hands of nurse | < LOQ | < LOQ | < LOQ | 0.02 |  |
| Nephrology | Nursing aide (face) | < LOQ | < LOQ | < LOQ | 0.03 |  |
| Nephrology | Nursing aide (hands) | < LOQ | < LOQ | < LOQ | < LOQ |  |
| Oncology | Face of nurse | 0.11 | 0.28 | < LOQ | 0.05 |  |
| Oncology | Arms of nurse | 0.03 | < LOQ | < LOQ | 0.03 |  |
| Haematology | Nursing aide 1 (face) | < LOQ | < LOQ | < LOQ | < LOQ |  |
| Haematology | Nursing aide 1 (hands) | < LOQ | < LOQ | < LOQ | < LOQ |  |
| Haematology | Nursing aide 2 (face) | 0.02 | < LOQ | < LOQ | < LOQ |  |
| Haematology | Nursing aide 2 (hands) | < LOQ | < LOQ | < LOQ | < LOQ |  |
| Haematology | Nursing aide 3 (face) | 0.02 | < LOQ | < LOQ | < LOQ |  |
| Haematology | Nursing aide 3 (hands) | < LOQ | < LOQ | < LOQ | < LOQ |  |
| Maternity oncology | Body samples of the nurse after activity (hands + face) | 0.03 | < LOQ | < LOQ | < LOQ |  |
| Maternity oncology | Body samples of psychologist after activity (hands + face) | 0.02 | < LOQ | < LOQ | 0.01 |  |

^a^CP = cyclophosphamide, IFO = ifosfamide, MTX = methotrexate, 5-FU = 5-fluorouracil
